# Supplementary material for: Rewritable ghost floating gates by tunnelling triboelectrification for two-dimensional electronics
Source: Nat Commun. 2017 Jun 26;8:15891. doi: 10.1038/ncomms15891 (PMC5490184; doi:10.1038/ncomms15891)
Supplement: Supplementary Information [file ncomms15891-s1.pdf]

File name: Supplementary Information

Description: Supplementary Figures, Supplementary Notes and Supplementary References

Supplementary Note 1. Mechanical stability of graphene after rubbing

The generation of triboelectric charges involves mechanical friction between the Pt-coated atomic force microscope (AFM) tip and graphene. In order to exclude damages to graphene, we analyzed topographic images of chemical vapor deposition (CVD) graphene after the rubbing process; Supplementary Fig. 1a shows a representative measurement. The white dashed box indicates the rubbed region. As evident, there is no detectable difference between the rubbed and unrubbed regions, consistently with the good mechanical durability of graphene[1, 2]. In addition, the cross section profile of Supplementary Fig. 1a along the blue dashed line shows no height difference between the two regions (Supplementary Fig. 1b). We conclude that our rubbing procedure does not result in significant damages for graphene.

Supplementary Note 2. Additional experiments on the tunneling triboelectrification mechanism

2.1 Tunneling triboelectrification by friction between the AFM tip and graphene

The potential difference between the unrubbed and rubbed region could in principle be ascribed to the friction, induced by the AFM tip, between the graphene layer and the SiO_2 layer. However, this possibility can be ruled out for the following reasons.

First, we could not obtain detectable potential differences between the rubbed and the unrubbed parts when using mechanically exfoliated graphene (MEG, see Fig. 2b and 2c), consistently with the tunneling mechanism (mechanically exfoliated graphene is clearly less defective than CVD graphene). By contrast, the hypothetical friction of mechanically exfoliated graphene with SiO_2 would likely give comparable (at least, for the orders of magnitude) results as the hypothetical friction of CVD graphene with SiO_2.

Second, the analysis of the triboelectric series for SiO_2, Pt and graphene reveals that our results may not be explained by the friction of graphene and SiO_2. In order to determine the relative positions of SiO_2 and Pt in the triboelectric series, we rubbed SiO_2 (300 nm) over a $2 \times 2\ \mu m^2$ area using a grounded Pt-coated AFM tip in contact mode and measured the surface potential ($5 \times 5\ \mu m^2$) both before and after triboelectrification, similar to the experiments shown in Fig. 1. Supplementary Fig. 3a shows the uniform surface potential of SiO_2 before rubbing. Supplementary Fig. 3b shows that, after rubbing, the surface potential of the rubbed region decreased contrastively to results in Fig. 1c which shows an increased surface potential in the rubbed region. As a consequence, Pt is positive with respect to SiO_2 in the triboelectric series. In case of friction between Pt and graphene (Supplementary Fig. 3d), the sign of the triboelectric charges is determined by the work functions (Φ) of the two materials. In practice, after friction, electrons transfer from graphene ($\Phi_{Graphene} \approx 4.5$ eV) to Pt ($\Phi_{Pt} \approx 5.9$ eV) and then, graphene becomes positive and Pt becomes negative. The resulting triboelectric series of SiO_2, Pt, and graphene is schematically described in Supplementary Fig. 3f, so that, in the hypothetical case of friction between SiO_2 and graphene, SiO_2 would become negative and graphene positive (Supplementary Fig. 3e), contrary to our experimental results.

In conclusion, both the absence of detectable effects when using mechanically exfoliated graphene and the triboelectric series of SiO_2, Pt, and graphene confirm that the localization of charges on the insulator underneath graphene is not induced by friction between graphene and SiO_2, but is determined by the tunneling of triboelectric charges generated by friction between the Pt-coated AFM tip and graphene.

2.2 Localization of charges at the air-SiO_2 interface

In principle, the triboelectric charges might also be trapped in the defects or impurities of CVD graphene rather than being stored at the air-SiO_2 interface. In fact, this is possible during a very short transient, but this mechanism may not justify the very slow decay of the potential difference between the rubbed and unrubbed graphene areas (e.g. see Fig. 1d, 1e and Supplementary Fig. 4). Additional charges trapped in graphene defects or impurities would create potential differences in graphene, but, since CVD graphene is not insulating, these potential differences would immediately result in currents which would tend to make graphene equipotential. In CVD graphene, different from insulators, these processes would be extremely fast (i.e. in conductors or semiconductors, significant potential differences may not be maintained for long times in absence of an external perturbation). In other words, after a very fast transient, potential differences across the CVD graphene (similar to other conductors or semiconductors) would quickly disappear.

By contrast, the existence, for very long times (e.g. many days), of potential differences in graphene is easily explained by the presence of electric charges localized on an insulator underneath graphene, similar to MOS (metal-oxide-semiconductor) capacitors, the difference being that our gate is floating, immaterial, and re-writable (i.e. time variant). The localization of charges at the air-SiO_2 interface is also confirmed by the accuracy of the equivalent circuit shown in Fig. 2d, which gives reasons of the presence of a shorter time constant (associated to the discharge of the oxide capacitor) and of a longer time constant (associated to the discharge of the air-gap capacitor) as well as of the higher magnitude of the slow-decay term (almost all the charges localize on the air-gap capacitor rather than on the SiO_2 capacitor, see main text). We mention that the localization of the charges at the air-SiO_2 interface is also confirmed by the Dirac point shift (Supplementary Note 4 and Supplementary Fig. 13).

2.3 Triboelectrification of graphene on different substrates

As an additional confirmation, in order to further verify that charges are trapped at the air-gap SiO_2 interface, we also carried out identical experiments with conductive metal substrates. The CVD graphene sheet was transferred on copper (Cu) substrates was treated by HF to remove the native oxide layer (Supplementary Fig. 5). Then, the top surface of graphene was rubbed with the Pt-coated AFM tip and the surface potential was measured using KPFM. In contrast with the case of insulating substrates, triboelectric charges were not localized in the rubbed region (white dashed square) but spread to the whole region (Supplementary Fig. 5c), thus confirming that the presence of an insulator under graphene is crucial.

Moreover, we also repeated the same experiments with CVD graphene deposited on other insulating substrates such as mica and Al_2O_3 (see Supplementary Fig. 6) and, similar to the case of CVD graphene on SiO_2, found that charges, after tunneling through CVD graphene, were trapped at the interface between air-gap and SiO_2.

Supplementary Note 3. Charges stored on two series capacitors

3.1 Fundamentals on charges stored on two series capacitors

In general, series capacitors may have a non-zero net charge on their two adjacent plates (a similar situation can be found in switched-capacitor circuits where, assuming the input op amp currents can be neglected, during some periods, two capacitors can be considered as in series even if the net charge on their two adjacent plates is non-zero). For this reason, with reference to the circuit shown in Supplementary Fig. 8 it is, in general, impossible to determine the DC voltages across C_1 and C_2 (e.g. circuit simulators such as SPICE would not be able to predict the DC voltage V_X in the circuit in Supplementary Fig. 8, but would simply

warn that node X is floating).

With reference to the circuit shown in Supplementary Fig. 8, the charges associated to the capacitor C_1 are $+Q_1$ and $–Q_1$ and, similarly, the charges associated to the capacitor C_2 are $+Q_2$ and $–Q_2$. In general, we may not assume $+Q_1$ and $+Q_2$ are identical (i.e. we may not assume the net charge on the adjacent plates of C_1 and C_2, i.e. $Q_2 – Q_1$, is zero). This is certainly possible and would, in fact, be the case if, at a certain instant t_X, V_B is equal to zero and both the capacitors are fully discharged (i.e. both $+Q_1$ and $+Q_2$ equal to zero); in such case, at t_X, the net charge on the adjacent plates of C_1 and C_2, i.e. $Q_2 – Q_1$, would obviously also be zero; therefore, due to the conservation of charge, if the voltage V_B changes, since, ideally (i.e. neglecting leakage currents), no charge can flow through dielectrics, the net charge on the adjacent plates of C_1 and C_2, i.e. $Q_2 – Q_1$, will be equal to zero for ever. As a result, in this specific case, by imposing $Q_2 = Q_1$ we would find the well-known relation

$$V_X = \frac{V_B C_1}{C_1 + C_2} \quad (1)$$

Such a relation is, however, not true if the net charge on the adjacent plates of C_1 and C_2, i.e. $Q_2 – Q_1$, is different from zero.

The following fictitious experiment, schematically represented in Supplementary Fig. 9, shows that the net charge on the adjacent plates of C_1 and C_2, i.e. $Q_2 – Q_1$, can be different from zero. For simplicity, we consider ideal capacitors (e.g. we neglect leakage), ideal switches (e.g. infinite off-resistance and zero on-resistance) and two given voltages (9 V and 1V, without any loss of generality as the same discussion applies for arbitrary voltages); moreover, we assume that C_1 and C_2 are identical ($C_1 = C_2$). With such assumptions, consider the following experiment:

a) C_1 is charged to 9 V by a 9 V DC voltage source (Supplementary Fig. 9a, switches S_{1A} closed and all the other switches opened)

b) C_1 is disconnected from the DC voltage source and C_2 is charged to 1 V by a 1 V DC voltage source (Supplementary Fig. 9b, switches S_{2B} closed and all the other switches opened); the voltage across C_1 will stay constant at 9 V because C_1, after disconnection from the 9 V voltage source, is in series with an open circuit ($i = 0$) and, therefore, according to the constitutive capacitor equation $i = C\frac{dv}{dt}$, its voltage must be constant and equal to 9 V

c) All the switches are opened (Supplementary Fig. 9c) so that both the capacitor voltages will stay constant at 9 V (C_1) and 1 V (C_2), respectively (both capacitors are connected in series with an open circuit)

d) The switch S_{3C} is closed (Supplementary Fig. 9d) and, therefore, connect in series the capacitors C_1 and C_2; during this step, at all times, each capacitor is in series with an open circuit and, therefore, the voltages across both C_1 and C_2 will stay constant and equal to 9 V and 1 V, respectively, which obviously corresponds to different values for the charges Q_1 and Q_2 (we assumed $C_1 = C_2$, so if the voltages are different, the charges are different), i.e. to a net charge on the adjacent plates of C_1 and C_2, i.e. $Q_2 – Q_1$, different from zero.

3.2 Distribution of charges after tunneling triboelectrification

With reference to the two small-area series capacitors in Fig. 2d, after tunneling triboelectrification, the net charge on the adjacent plates of the two capacitors (i.e. the tunneling triboelectric charges trapped in the insulator) is certainly not zero. In fact, the existence of a significant potential difference within the graphene layer for very long times

(Fig. 1) is only possible in presence of localized charges trapped in an insulator (localized charges in both silicon and graphene would not stay localized for long times and would quickly spread out). As a result, since the net charge on the adjacent plates of the two capacitors is not zero, we may not use the relation $V_X = \frac{V_B C_1}{C_1 + C_2}$ in order to compute how the charges distribute across the small-area air gap capacitor and the small-area SiO_2 series capacitor.

However, it is easy to see that almost all the tunneling triboelectric charges, Q_{TT} (i.e. a fraction, which may be close to one in case of 1L CVD graphene, of the triboelectric charges), are stored on the small-area air gap capacitor. In fact, after tunneling through graphene, the charges Q_{TT} are trapped at the interface between air and SiO_2 and, therefore, electrostatically attract an equal amount (magnitude) of charges of the opposite type on the graphene layer and/or on the silicon underneath silicon oxide. In other words, with reference to Fig. 2d, since charges may not travel through dielectrics, the charges Q_{TT} must be stored on the top plate of the SiO_2 small-area capacitor and/or on the bottom plate of the small-area air capacitor. However, as graphically illustrated in Fig. 2d, almost all these opposite charges are attracted from graphene because of the much smaller thickness (i.e. larger capacitance) of the small-area air capacitor in comparison with the small-area SiO_2 capacitor. In fact, in order to determine the steady state (after transient) distribution of charges, only capacitors must be considered (at DC capacitors behave as open circuits which, in series with a conductive path, such as a graphene layer, dominate the impedance). Moreover, the large-area capacitors are much bigger than the small-area capacitors interested by the friction process and, therefore, dynamically behave as short circuits (i.e. the large area capacitors may be approximately seen as infinite capacitances, i.e. ideal voltage sources, i.e. equivalent, from a dynamic point of view, to short circuits). As a result, with reference to Fig. 2d, the charge Q_{TT} is shared

between the small-area air capacitor and the small-area SiO_2 capacitor as it would be shared if these two capacitors were in parallel (as both these capacitors have a terminal which, dynamically, is grounded) and therefore, similar to charge sharing in parallel capacitors, most charges accumulate across the larger capacitance (i.e. the small-area air capacitor). As a consequence, the charges stored on the small-area SiO_2 capacitor are almost unaffected by tunneling triboelectrification and, therefore, the voltage drop across the small-area SiO_2 capacitor is also almost unchanged (i.e. stays at zero) so that the voltage drop measured across graphene by KPFM is almost identical to the voltage drop across the small-area air gap capacitor.

As a result, by considering the air gap thickness t_{Air} (Supplementary Fig. 2), we easily estimate the charge density (Fig. 3e) stored underneath graphene in (x,y) as $\dfrac{\varepsilon_{Air}\Delta V_{TT}\left(x,y\right)}{t_{Air}}$ where ΔV_{TT} is the surface potential measured (by KPFM) in the point (x,y) taken with reference to the average surface potential of the unrubbed region (see main text), $\varepsilon_{\mathrm{Air}}$ is the dielectric constant of air and t_{Air} is the thickness of air gap.

3.3 Decay time of ΔV_{TT}

In the hypothetical circuit shown in Supplementary Fig. 10a, the two capacitors C_1 and C_2 are in parallel (each capacitor connects the same couple of terminals). The two parallel capacitors C_1 and C_2 could, therefore, also be represented as a single capacitor with capacitance C_1+C_2; as a result the discharge of the capacitors would be exponential with a single time constant equal to $(R_1//R_2)(C_1+C_2)$, where $R_1//R_2$ is the parallel of R_1 and R_2, i.e. $R_1R_2/(R_1+R_2)$.

However, in case of tunneling triboelectrification, after the equilibrium has been reached, there is no current across graphene, thus resulting in the equivalent circuit shown in

Supplementary Fig. 10b. As a consequence, due to Kirchoff's current law (i.e. conservation of charge), there may be no current going from the top bipole (parallel connection of R_1 and C_1) to the bottom bipole (parallel connection of R_2 and C_2). Therefore, the capacitor C_1 may only discharge through the resistor R_1, thus resulting in a time constant R_1C_1; similarly, the capacitor C_2 may only discharge through the resistor R_2, thus resulting in a time constant R_2C_2, in perfect agreement with our experiments (e.g. see the excellent agreement between experimental points and the best fit in Fig. 1e). As a result, the total voltage (measured by KPFM) will evolve with time as

$$v(t) = V_1 e^{\frac{-t}{\tau_1}} + V_2 e^{\frac{-t}{\tau_2}} \tag{2}$$

where V_1 is the initial voltage (at $t = 0$) across C_1, V_2 is the initial voltage (at $t = 0$) across C_2, $\tau_1 = R_1C_1$, and $\tau_2 = R_2C_2$.

In practice, in case of tunneling triboelectrification, one of these two addends dominates at all times as it has both a higher initial magnitude and a longer time constant. In fact, the higher initial magnitude is certainly associated to the air gap capacitor because almost all the tunneling triboelectric charges are localized across the air gap capacitor (see main text and Supplementary Note 3.2); additionally, air is a much better insulator than silicon oxide and, therefore, the time constant associated to the air gap capacitor is much longer than the time constant associated to the SiO_2 capacitor.

These results are in perfect agreement with all our experiments and give reasons for the exceptionally long time constants we found after tunneling triboelectrification (more than two orders of magnitude longer than for conventional triboelectrification of a dielectric).

Supplementary Note 4. Control of CVD graphene devices by ghost floating gates

4.1 Resistance control on CVD graphene resistor

Supplementary Fig. 12 shows the resistance reduction induced on the 4-contacts 1L CVD graphene resistor shown in Fig. 4. In practice, as schematically shown in Supplementary Fig. 12a, we performed 4-wires measurements by injecting a 100 nA current through the force electrodes F_1 and F_2 and measuring the voltage difference across the inner sense electrodes S_1 and S_2 (the instrumentation amplifier *IA* has negligible input currents and, therefore, first, the entire I_0 current flows through the graphene resistor R_G, and, second, the voltage across the input terminals of the instrumentation amplifier is exactly the same as the voltage across R_G). For instance, Supplementary Fig. 12b-d show the voltages across R_G (i.e. 100 nA × R_G) before (higher value of R_G and, therefore, of the voltage difference across R_G) and after rubbing the middle area (yellow dashed box, between the sense electrodes S_1 and S_2 in Fig. 4a) with an AFM-Pt tip biased at -10 V (b), -5 V (c), and 0 V (d), respectively. As evident, the tunneling triboelectric charges act as ghost floating gates and electrostatically reduce the normalized resistance R/R_0 (where R is the resistance of graphene after rubbing and R_0 is the resistance before rubbing). These results already constitute a device-level demonstration of tunneling triboelectrification (e.g. tunable resistors are useful in tunable amplifiers, automatic gain control circuits, tunable voltage/current references, etc.).

4.2 Dirac point shift by tunneling triboelectrification

In addition to the control of graphene resistivity by tunneling triboelectrification (Fig. 4 and Supplementary Fig. 12), we also verified that tunneling triboelectrification allows to control the Dirac point of graphene (Supplementary Fig. 13). In practice, we measured the drain-to-source current (I_{DS}) of a graphene resistor as a function of the back gate voltage (V_{BG}) both

before and after rubbing p-doped graphene with an AFM tip biased at +10 V. The I_{DS}-V_{BG} curves were measured under constant drain-to-source voltage (V_{DS} = 0.01 V). As described in Supplementary Fig. 13b, after tunneling triboelectrification, the Dirac point of the graphene resistor shifts to the left and, with a zero gate voltage, the effective doping turns from p-type to n-type. In fact, consistently with our theoretical discussions, first, positive charges are generated in graphene by triboelectrification with the positively biased AFM tip and, second, part of these positive charges tunnel through the air gap and effectively turn the naturally p-type graphene into n-type (Supplementary Fig. 13a). This situation is exactly the same found in MOS (metal-oxide-semiconductor) capacitors where the presence of charges on the metal gate induces opposite charges on the semiconductor and, therefore, travelling through the semiconductor at the semiconductor-oxide interface, going from the region underneath the gate to the surrounding regions of the semiconductor, there are potential differences (due to the gate charges) which may be well preserved for very long times. This is exactly analogous to our case, the difference being that our gate is floating, immaterial, and re-writable (or, equivalently, time variant). In conclusion, Supplementary Fig. 13b confirms that tunneling triboelectrification can effectively control the current transport characteristics of 2D devices.

We mention that Supplementary Fig. 13b also provides an additional confirmation that the positive charges induced on graphene by rubbing with the positively biased AFM tip (+10 V) tunnel through the air gap and localize at the SiO_2-air interface; in fact, such localized positive charges electrostatically attract free charges of the opposite type (negative) in graphene (i.e. turn graphene from p-type to n-type doping), similar to what would happen with positive charges localized on an hypothetic gate underneath the air-gap.

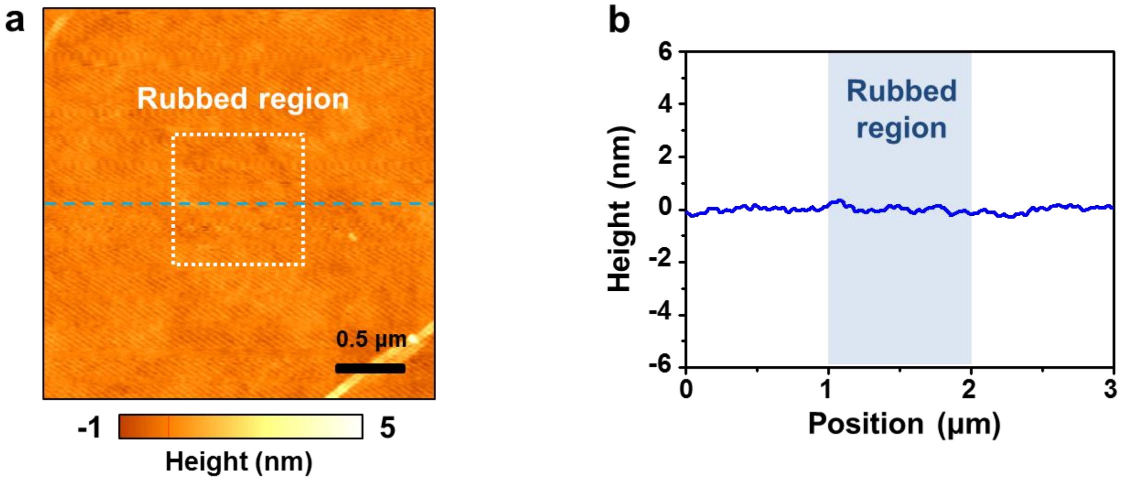

Supplementary Figure 1 | Absence of damages in CVD graphene after rubbing with the Pt AFM tip. (**a**) Topography image of CVD graphene after rubbing. The white box indicates the rubbed region. (**b**) Cross-sectional profile along the blue dashed line in (**a**).

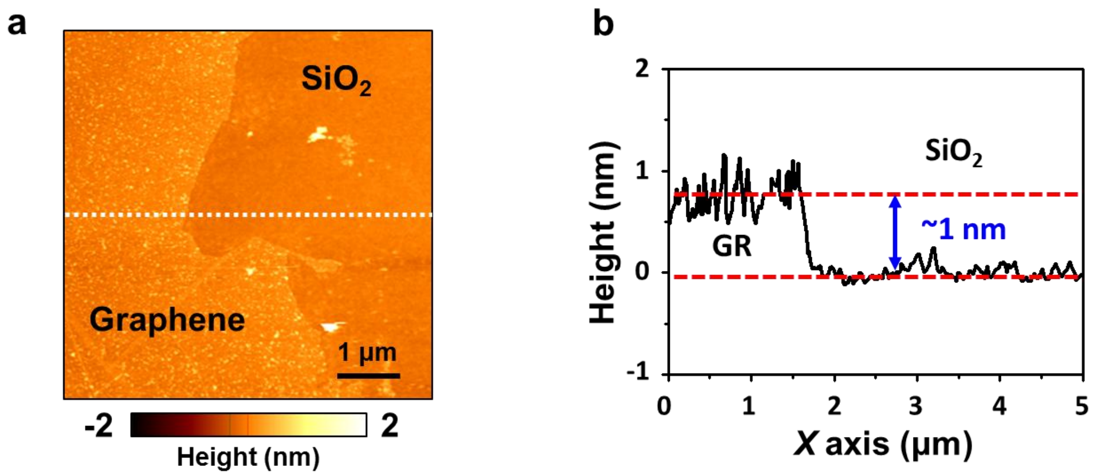

Supplementary Figure 2 | AFM measurement of the air gap thickness. The step is about 1 nm, so that, taking into account the 0.34 nm thickness of monolayer graphene, the air gap thickness can be estimated around 0.66 nm. (**a**) Topography image of CVD graphene on SiO_2. (**b**) Cross-sectional profile along the white dashed line in (**a**).

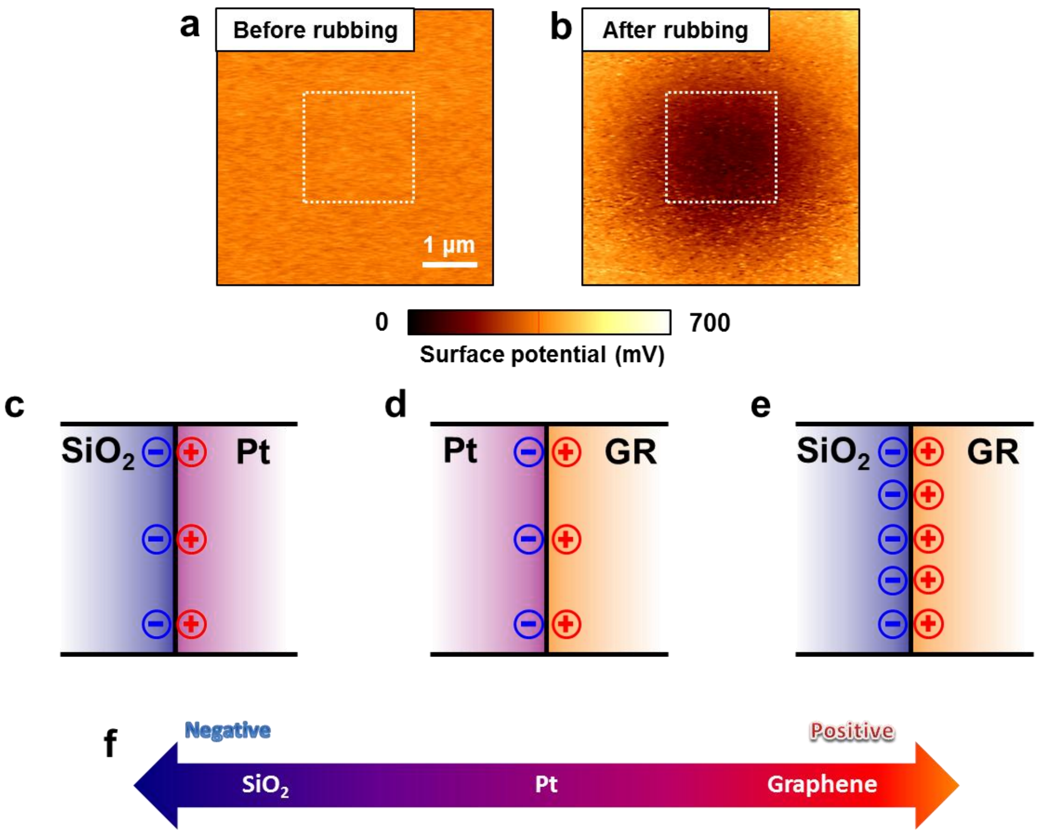

Supplementary Figure 3 | Triboelectric series of SiO_2, Pt and graphene. (**a**) KPFM image of SiO_2 before and (**b**) after rubbing with Pt-coated AFM tip. (**c**)-(**e**) Schematic representation of the triboelectric charge transfer processes in (**c**) SiO_2/Pt, (**d**) Pt/graphene and (**e**) SiO_2/graphene. (**f**) Triboelectric series of SiO_2, Pt and graphene.

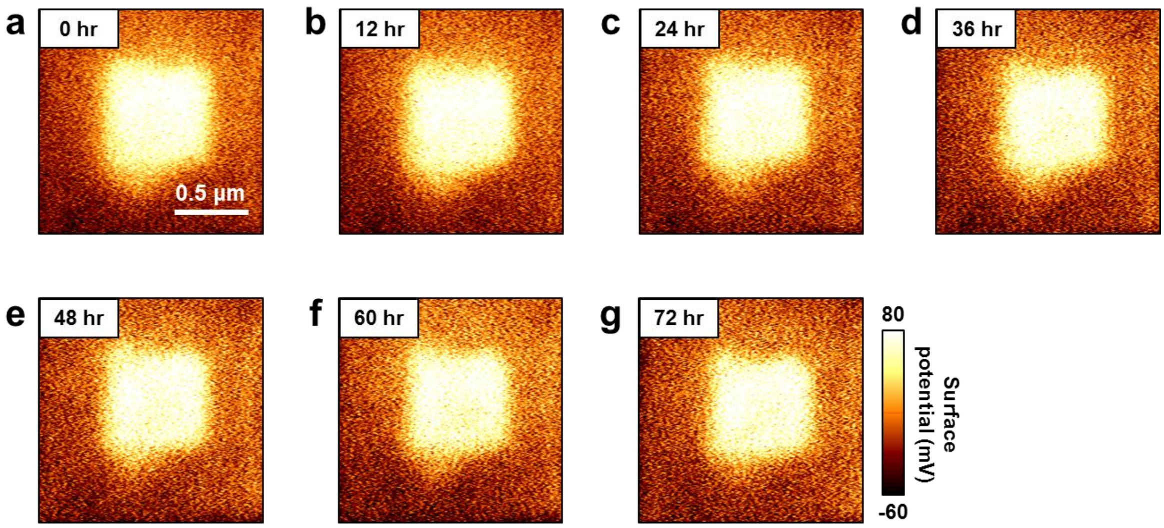

Supplementary Figure 4 | KPFM images taken at different times after rubbing 1L CVD graphene. (**a**)-(**g**) The localized triboelectric charges were very well preserved under the rubbed region even after 72 hours.

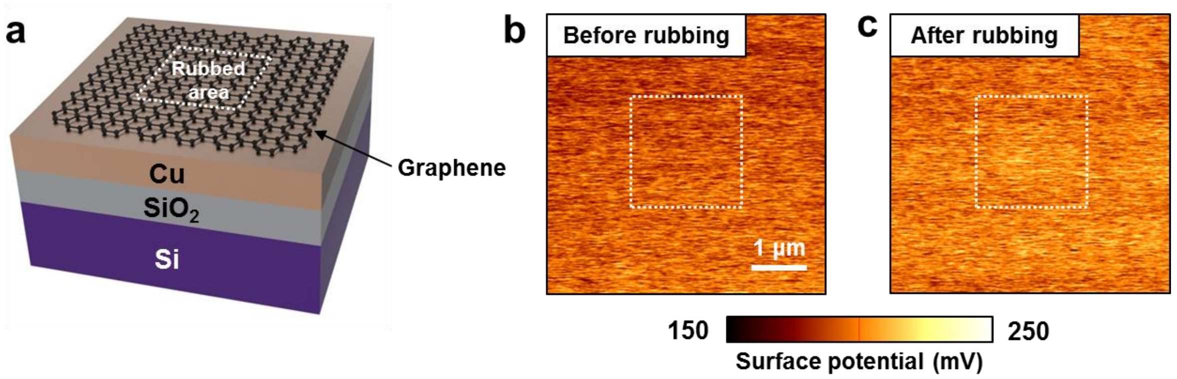

Supplementary Figure 5 | Triboelectrification of graphene on metal substrate. (**a**) Schematic image of graphene transferred on metal (Cu) substrate. (**b**) KPFM image of graphene on metal substrate before rubbing and (**c**) after rubbing with Pt-coated AFM tip.

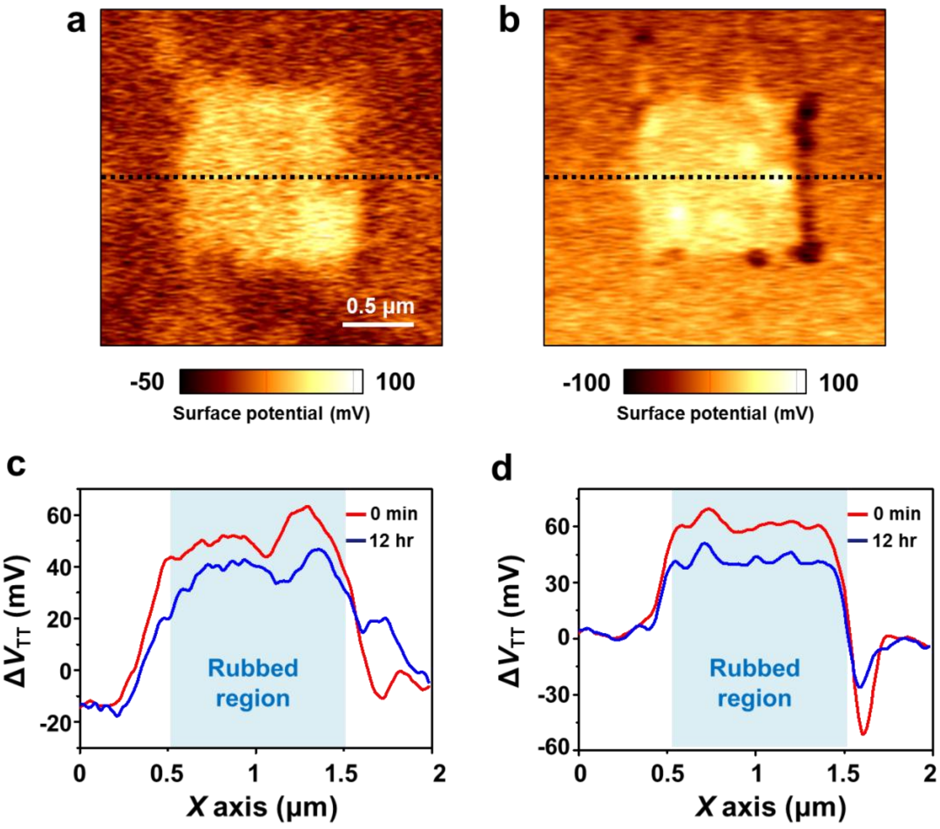

Supplementary Figure 6 | Tunneling triboelectrification of graphene on mica and on Al_2O_3. (**a**), (**b**) KPFM images, after tunneling triboelectrification, of CVD graphene deposited on (**a**) Mica and CVD graphene deposited on (**b**) Al_2O_3. (**c**), (**d**) Cross-sectional profiles along the black dashed lines for graphene on (**c**) Mica and graphene on (**d**) Al_2O_3 after 0 min and 12 hours.

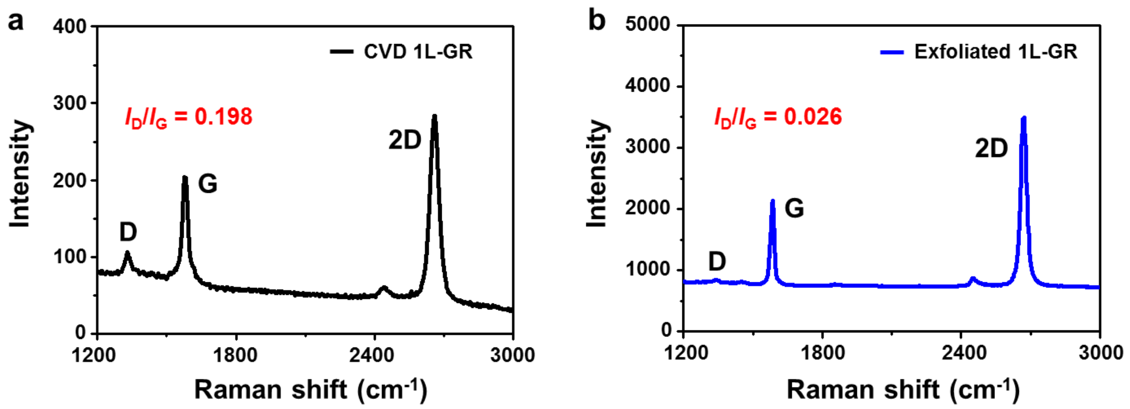

Supplementary Figure 7 | Raman spectra of 1L CVD graphene and mechanically exfoliated 1L graphene. (**a**) Raman spectra of 1L CVD graphene. (**b**) Raman spectra of exfoliated 1L-graphene, showing D, G, 2D peaks at ~ 1300, ~ 1600 and ~ 2700 cm^{-1} respectively. The ratios of I_G/I_{2D} = ~ 0.5 in (**a**) and (**b**) show that they are mono-layer graphene. The ratios of I_D/I_G = 0.198, 0.026 for CVD graphene and exfoliated graphene, respectively, show that the CVD graphene has a lot of defect regions compared to the exfoliated graphene (Renishaw, RM-1000 Invia, 514 nm, Ar^+ ion laser).

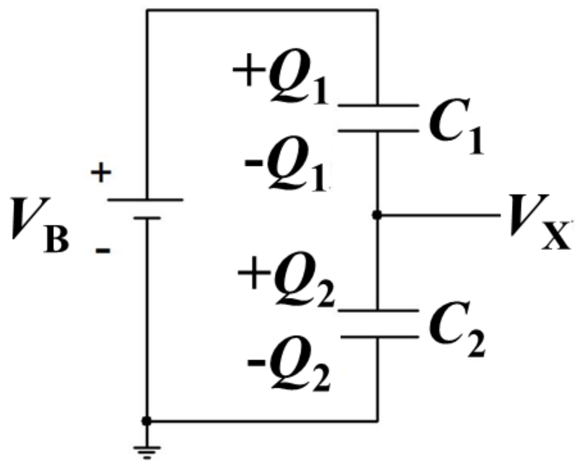

Supplementary Figure 8 | Circuit for illustrating how charges distribute on two capacitors in series.

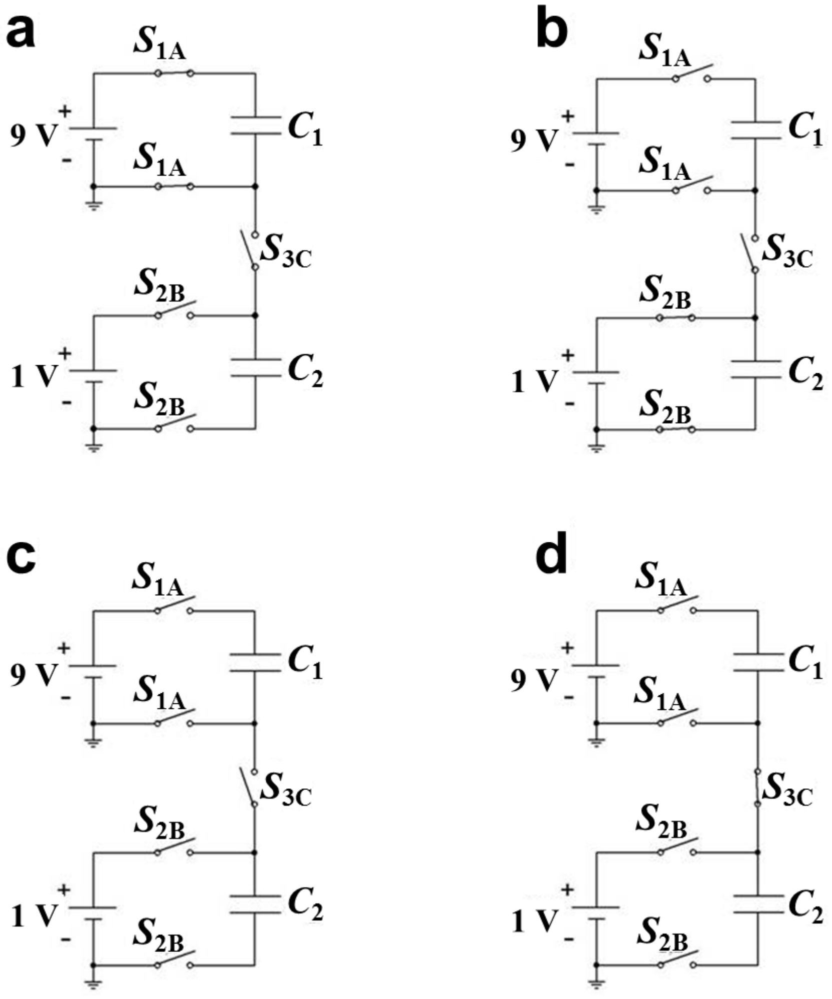

Supplementary Figure 9 | Fictitious experiment illustrating that two arbitrary charges can be stored on two series capacitors. (**a**) C_1 is charged at 9 V. (**b**) C_2 is charged at 1 V, the voltage across C_1 is constant because $i_{C1} = C\frac{dv_{C1}}{dt} = 0$. (**c**), (**d**) The voltages across C_1 and C_2 are constant because $i_{C1} = C\frac{dv_{C1}}{dt} = 0$ and $i_{C2} = C\frac{dv_{C2}}{dt} = 0$, respectively.

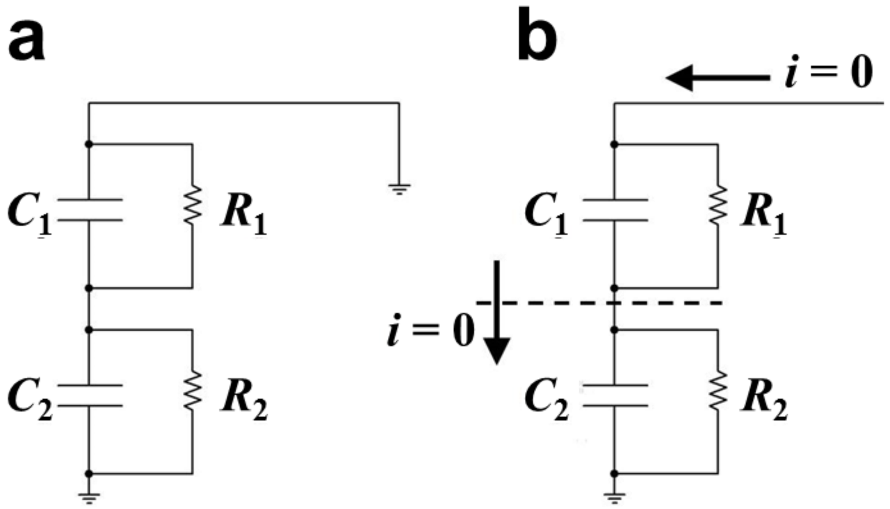

Supplementary Figure 10 | Discharge of capacitors in different configurations. (**a**) For two capacitors in parallel, each one with a resistance in parallel, the decay is exponential with a single time constant equal to $(\boldsymbol{R}_1 // \boldsymbol{R}_2)(\boldsymbol{C}_1 + \boldsymbol{C}_2)$. (**b**) Equivalent circuit for the decay of the voltage ΔV_{TT}; since the current through the graphene layer is zero, each capacitor exclusively discharges through its parallel resistor, resulting in two distinct time constants $\boldsymbol{R}_1\boldsymbol{C}_1$ (associated to the air gap capacitor) and $\boldsymbol{R}_2\boldsymbol{C}_2$ (associated to the SiO_2 capacitor).

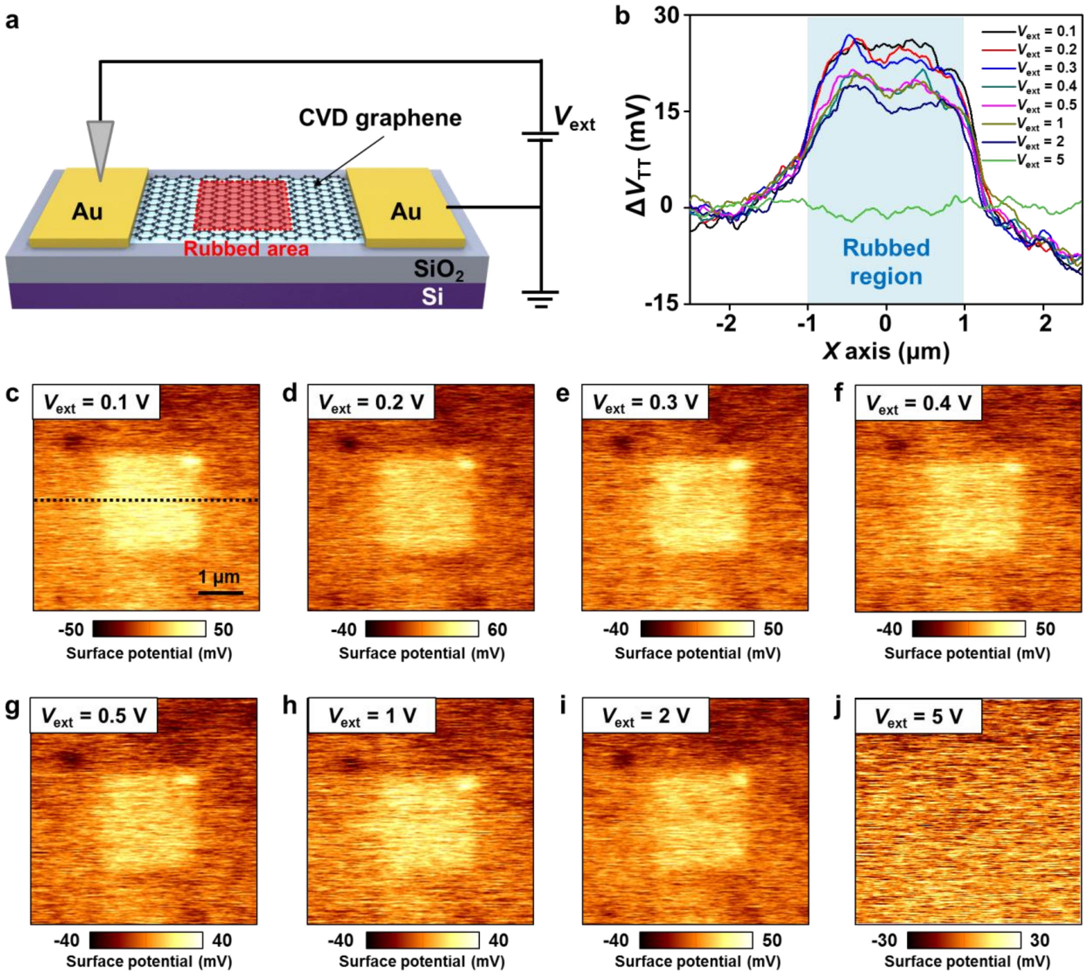

Supplementary Figure 11 | Trapped charges survive even under relatively high currents flowing through the rubbed graphene. (**a**) Schematic image of the device structure; after rubbing, the AFM tip is used to apply an external voltage for 7 minutes and induce currents in the mA range. (**b**) Cross-sectional profiles along the black dashed line in (**c**) to (**j**). These profiles clearly show the remaining of tunneling triboelectric charges even after applying the external voltage up to a certain threshold. (**c**) to (**j**) KPFM images after applying an external voltage V_{ext} (0.1 ~ 5 V) on CVD graphene which has localized charges under graphene (bright region in the center of the CVD graphene). The localized tunneling triboelectric charges were well preserved after applying V_{ext} = 2 V, but were completely erased after applying V_{ext} = 5 V.

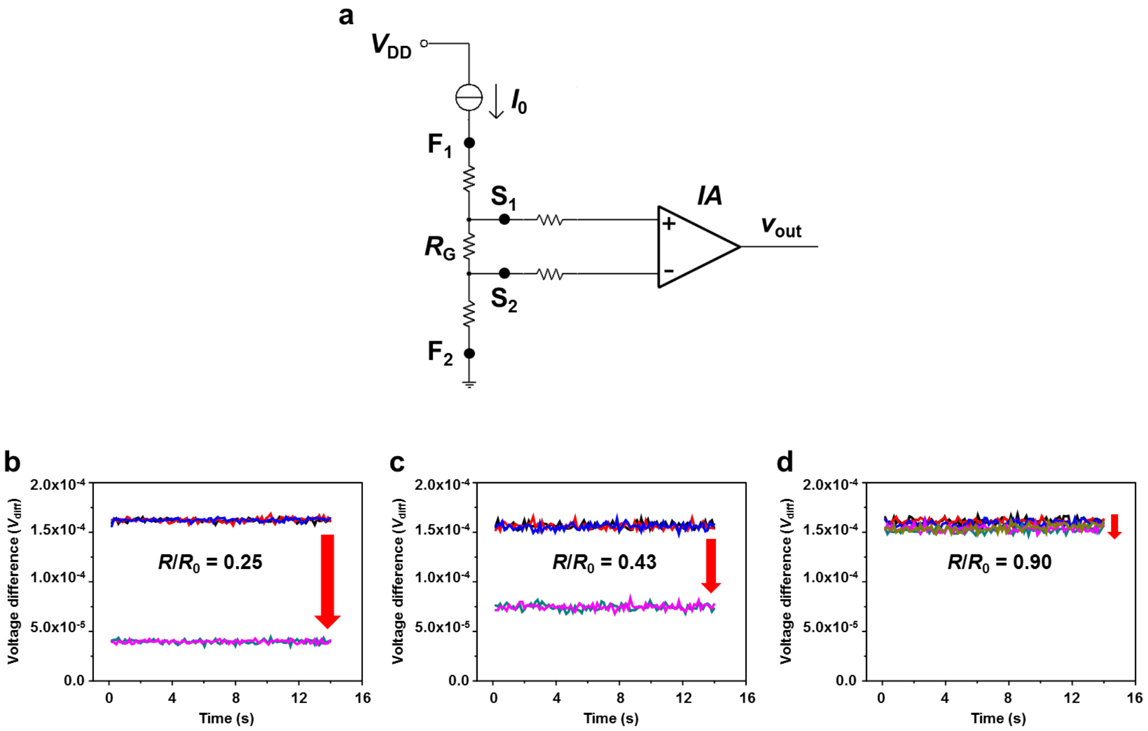

Supplementary Figure 12 | Resistance reduction of graphene by tunneling triboelectrification. (**a**) Schematic representation of 4-contacts 1L CVD graphene resistor and of the 4-wires measurement technique. (**b**)-(**d**) Voltages measured across the sense terminals S_1 and S_2 with I_0 = 100 nA before and after AFM tunneling triboelectrification performed with tip bias voltages of (**b**) -10 V, (**c**) -5 V, (**d**) 0 V.

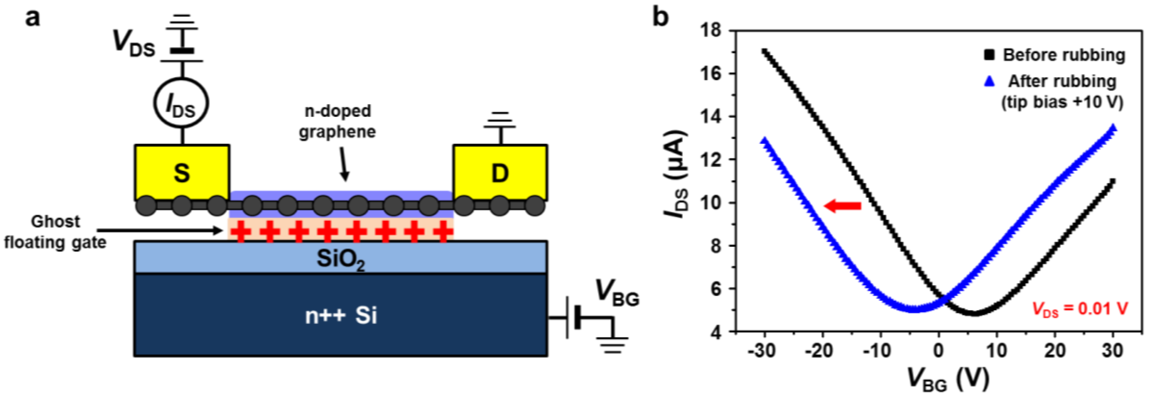

Supplementary Figure 13 | Control of the Dirac point by tunneling triboelectrification. (**a**) Schematic image of the device structure and of the ghost floating gate. (**b**) I_{DS}-V_{BG} curves before (black) and after (blue) tunneling triboelectrification (V_{DS} = 0.01 V).

Supplementary References

1. Lee, C., Wei, X., Kysar, J. W. & Hone, J. Measurement of the elastic properties and intrinsic strength of monolayer graphene. *Science* **321,** 385-388 (2008).

2. Geim, A. K. Graphene: status and prospects. *Science* **324,** 1530-1534 (2009).
